# The impact of TNF superfamily molecules on overall survival in acute myeloid leukaemia: correlation with biological and clinical features

**DOI:** 10.1007/s00277-014-2178-x

**Published:** 2014-08-03

**Authors:** L. Bolkun, D. Lemancewicz, E. Jablonska, A. Szumowska, U. Bolkun-Skornicka, W. Ratajczak-Wrona, J. Dzieciol, J. Kloczko

**Affiliations:** 1 Department of Haematology, Medical University of Bialystok, 24a Sklodowskiej-Curie, Bialystok, 15-276 Poland; 2 Department of Human Anatomy, Medical University of Bialystok, Bialystok, Poland; 3 Department of Immunology, Medical University of Bialystok, Bialystok, Poland; 4 Department of Pharmaceutical Technology, Medical University of Bialystok, Bialystok, Poland

**Keywords:** Acute myeloid leukaemia, APRIL, BAFF, TRAIL

## Abstract

B cell-activating factor (BAFF), a proliferation-inducing ligand (APRIL) and apoptosis-inducing ligand (TRAIL) were demonstrated in several haematological diseases including acute myeloid leukemia (AML). Those cytokines are capable of activating a broad spectrum of intracellular signalling cascades that can either induce apoptosis or protect from programmed cell death. We have analysed BAFF, APRIL and TRAIL serum concentrations in 76 patients with newly diagnosed AML and 40 healthy volunteers. The values were significantly higher for APRIL and BAFF but lower for TRAIL compared to healthy volunteers. Induction therapy significantly reduced the values for BAFF and increased them for TRAIL. Moreover, the concentration of BAFF and APRIL was significantly lower and the concentration of TRAIL higher in a group of patients with complete remission compared to non-respondent AML patients. In addition, higher concentrations of BAFF and lower of TRAIL predicted a shorter overall survival, suggesting thereby an important prognostic marker and possible therapeutic target in AML.

## Introduction

Acute myeloid leukaemia (AML) represents a group of clonal haematopoietic stem cell disorders in which both failure to differentiate and overproliferation in the stem cell compartment result in the accumulation of non-functional myeloid cells termed myeloblasts and loss of normal haematopoietic function. Considerable effort has been invested in identifying prognostic markers that might predict clinical outcomes in AML patients. There are currently several characteristics, such as advanced age, cytogenetic and performance status, that are commonly used as predictors of survival [1, 2].

Two decades ago, the existence of receptors activated by the tumour necrosis factor alpha (TNF-α) was demonstrated for several haematological diseases, including AML cells [3, 4]. Importantly, there are different effects of the TNF-α which can be observed in AML blasts. The cytokine TNF-α is capable of activating a broad spectrum of intracellular signalling cascades that can either induce apoptosis (e.g. activation of caspase-8) or protect various cell types from programmed cell death (e.g. nuclear factor-kappa B (NF-κB) pathway activation) [5]. NF-κB activation is predominantly responsible for the expression of anti-apoptotic genes. Nevertheless, some proapoptotic genes, including Fas ligand or TRAIL, TNF-κ and p53, are targeted by NF-κB too [6].

TNF-related apoptosis-inducing ligand (TRAIL) molecule, which belongs to the TNF family of proteins, participates in the elimination of neoplastic cells, including leukaemic cells, in 60 % of cases [7, 8]. TRAIL, also known as Apo-2L, can be biologically effective as an integral membrane protein (mTRAIL, 32 kDa), as well as a soluble cytokine (sTRAIL, 24 kDa) [8]. Several important functions of TRAIL-induced apoptosis have been reported. First, TRAIL-mediated cytotoxicity plays an important role in innate and adaptive immune responses [9]. Second, TRAIL exerts a regulatory function on erythroid and myeloid maturation in normal haematopoiesis [10–13]. Moreover, senescent neutrophils are eliminated by TRAIL-induced apoptosis upon their return to the bone marrow [14].

The anti-tumour activity of TRAIL has been investigated in haematological malignancies, including multiple myeloma cells and Philadelphia chromosome-positive leukaemia, in which TRAIL was shown to be able to induce apoptosis [15, 16]. A few studies, carried out on a limited number of cases, exhibited a very low sensitivity of AML blasts to the apoptotic effects of TRAIL [17]. By contrast, some continuous cell lines derived from AML were observed to have a pronounced sensitivity to the TRAIL-mediated apoptotic effects [18].

B cell-activating factor (BAFF) and a proliferation-inducing ligand (APRIL), both members of the TNF family, produce an effect opposite to that of TRAIL. They represent two of the main survival factors for immature, naive and activated B cells [19]. BAFF, APRIL and TRAIL molecules were also detected in monocytes/macrophages, dendrite cells and activated T lymphocytes [7, 20]. Another significant source of these molecules is neutrophils, which participate in the early stage of anti-cancer response [21]. BAFF and APRIL are also known to directly activate the NF-κB pathway [22, 23]. There is some evidence that this pathway activity is also observable in the CD34+ AML cells [22]. Consequently, the inhibitors of NF-κB have emerged as potential therapies against AML. Depending on the B cell maturation stage, BAFF was reported to induce the anti-apoptotic proteins Bcl-2, A1 and Bcl-xL and to reduce the proapoptotic protein Bak [24, 25]. The not yet fully understood role of BAFF, APRIL and their receptors in normal B cell homeostasis and in several tumour models raises the possibility of their involvement in the pathogenesis of haematological malignancies and solid tumours.

Indeed, it has been demonstrated that APRIL mRNA and its protein, including its secreted form, are expressed in the leukaemic cells of patients with AML subtypes but not in normal haematopoietic progenitors. Retrovirus-mediated APRIL expression in normal haematopoietic progenitors confers resistance to the chemotherapeutic drug-induced apoptosis. Conversely, blocking APRIL function by recombinant soluble APRIL receptors increased the chemotherapeutic drug-induced cell death in AML cells [26].

The purposes of the present study were to evaluate serum levels of BAFF, APRIL and TRAIL in healthy volunteers and in AML patients with varying severity of the disease in order to determine whether there was any correlation between BAFF, APRIL and TRAIL at diagnosis and some prognostic biological parameters of AML patients and to explore their clinical significance in predicting the disease activity of AML.

## Patients

Seventy-six patients with newly diagnosed acute non-promyelocytic leukaemia were included in the study. Their median age at the time of sample collection was 45.5, and the range was 18–62. Thirty-nine subjects were female and 37 male. Both patients with acute promyelocytic leukaemia, due to the specific biology and a different outcome, as well as AML patients who received corticosteroids at the beginning of the treatment course had been excluded from the study. Diagnoses were established following the WHO classification system [27]. Blood counts and flow cytometry were performed in order to confirm the presence of blastic cells, whereas cytogenetic and molecular studies, including the FISH study (AML1/ETO, CBFß/ MYH11, MLLT3-MLL and frequently mutated genes FLT3-ITD, NPM1, CEBPA), determine the risk group, according to WHO recommendation. On the basis of the above, patients were classified as follows: 13 (17 %) patients had good risk (10 patients with *t*(8;21), 2 with inv(16) and one with mutated core-binding factor leukaemia (CEBPA_*mut*_), 17 (22.5 %) subjects had first intermediate risk (diploid karyotype features with 4 both mutated nucleophosmin (NPM1_*mut*_) and internal tandem duplication of Fms-like tyrosine kinase 3 (FLT3-ITD) and 9 with FLT3-ITD without NMP1_*mut*_), 13 (17 %) patients had second intermediate risk (3 patients with *t*(9;11) and 10 with different abnormalities not assigned to either good or bad risk group) and 33 subjects (43.5 %) were classified as unfavourable risk group with del(5q), del(7q) or complex (≥3) abnormalities. AML patients were treated in the Haematology Department of the Medical University of Bialystok from 2007 to 2012 with 7-day induction chemotherapy regimens corresponding to the standard therapy based on the Polish Adult Leukaemia Group: Cytarabine was delivered as a continuous IV infusion for seven consecutive days at a dose of 200 mg/m^2^, while anthracycline for three consecutive days as an IV push at a dose of 50 mg/m^2^, cladribine was administered for 5 days as an IV push at a dose of 5 mg/m^2^ (DAC schedule) [28]. After induction, the morphology response was evaluated following the recommendation by Chason et al. [29]; 44 patients achieved complete remission (CR) after first induction and 27 were non-responders (NR). Among all patients included in the study, there were no patients with partial remission. Patients who did not achieve CR were given re-induction (CLAG-M or/and ICE). All patients who achieved CR after first/second/third induction received two consolidation therapies: first HAM (cytarabine and mitoxantrone) and second HDAraC (high dose of cytarabine), prior to allogeneic stem cell transplantation (50 patients) or maintenance therapy in the case of patients with favourable cytogenetic risk (9 patients). The mortality during first/second/third inductions and consolidations stood at 5/5/3/0 patients. The characteristic of the patients is listed in Table 1.

**Table 1:** Characteristics of patients with AML

Characteristic	AML
Number of patients	76
Mean (range) age, year	45.5 (18–62)
Mean (±SD) white blood cell count (G/l)	32.01 ± 28.04
Mean (range) of the blastic cells in peripheral blood (range)	49 (0–93)
Mean (range) of blastic cells in bone marrow (range)	55 (20–98)
Acute myeloid leukaemia with recurrent genetic abnormalities	*n* (%)
*t*(8;21) (q22;q22);(AML1/ETO)	10 (13.1 %)
inv(16) (p13;q22) or *t*(16;16) (p13;q22); (CBFß/ MYH11)	2 (2.6 %)
*t*(9;11); MLLT3-MLL	3 (4.0 %)
AML with multilineage dysplasia without antecedent MDS	1 (1.3 %)
AML therapy-related	0 (0.0 %)
AML not otherwise categorized	61
AML, minimally differentiated	8 (10.5 %)
AML without maturation	13 (17.1 %)
AML with maturation	20 (26.3 %)
Acute myelomonocytic leukaemia (AMMoL)	14 (18.4 %)
AMMoL with eosinophilia	0 (0.0 %)
Acute monoblastic leukaemia	2 (2.6 %)
Acute monocytic leukaemia	2 (2.6 %)
Acute erythroid leukaemia	1 (1.3 %)
Acute megakaryoblastic leukaemia	0 (0.0 %)
Outcome of induction therapy, *n*	
CR achieved after first induction	44
CR achieved after second induction	12
CR achieved after third induction	3
Mortality during first/second/third induction/consolidation	5/5/3/0
Post-consolidation treatment	
AlloHSCT	50
Maintenance	9
*FLT3-ITD/NMP1*_*mut*_ */CEBPA*_*mut*_	13/4/1
Favorable risk	13 (17.0 %)
Intermediate risk I	17 (22.5 %)
Intermediate risk II	13 (17.0 %)
Unfavourable risk	33 (43.5 %)

*AML* acute myeloid leukaemia, *CR* complete remission, *AlloHSCT* allogenic haematopoietic stem cell transplantation, *NPM1*_*mut*_ mutated nucleophosmin, *CEBPA*_*mut*_ mutated core-binding factor leukaemia, *FLT3-ITD* internal tandem duplication of Fms-like tyrosine kinase 3

The control group was population-based and comprised forty age-matched healthy volunteers (20 males, 20 females). Samples were collected from the subjects that provided they had not had fever for 1 week, were not receiving any medications, were not be pregnant and did not have a history of any chronic diseases.

All patient samples, as well as samples from the healthy volunteers, were collected under the Ethics Committee of the Medical University of Bialystok upon signing an approved protocol and a written informed consent form in accordance with the Declaration of Helsinki, No. R-I-002/218/2007.

## Methods

The cytokine was collected at the time of diagnosis (76 patients) and after induction (71 patients), approximately 28 ± 2 days after the beginning of treatment. The measurement was done following the manufacturers’ instructions. Four independent sets of experiments were performed. Each experiment included the kit’s standards run in triplicate and samples both from the AML patients and healthy controls. No significant variations were observed among the experiments. Quantitative assessments of cytokines were performed by means of ELISA assays*.* Soluble APRIL concentrations were measured in the serum with Human APRIL (Platinum ELISA, eBioscience Austria). Soluble TRAIL, BAFF, IL-6 and TNF were evaluated in the serum using commercially available test kits (Quantikine®, R&D kits, Minneapolis, MN, USA).

## Statistics

Results were expressed as median (range). The chi-square or, alternatively, the Kruskal–Wallis test was used to assess relationships between categorical variables. Comparisons between the AML and the control groups were made using the non-parametric Mann–Whitney test. The Spearman’s order correlation coefficient was applied to determine correlations between the measured parameters. Survival times were compared by means of the log-rank test. Multivariate models were reduced by one factor at a time to ensure that all the factors remaining in the model were statistically significant at the 5 % level. *p* Values below 0.05 were considered to be statistically significant. Descriptive statistics were analysed, and univariate analyses were performed.

## Results

### Serum APRIL, BAFF, TRAIL, TNF-α and IL-6 concentration

Median (range) serum values of BAFF, APRIL, TRAIL, IL-6, TNF-α of AML patients and healthy volunteers are listed in Table 2. Pre-treatment AML patients had a significantly higher serum concentration compared to healthy volunteers for BAFF, 3,651.1 pg/ml (983.9–9151.4 pg/ml) versus 651.4 pg/ml (362.9–1,122.3 pg/ml), *p* < 0.0001; APRIL, 5.96 ng/ml (1.65–43.4 ng/ml) versus 1.68 ng/ml (1.0–7.56 ng/ml), *p* < 0.0001; IL-6, 12.12 pg/ml (1.96–43.4 pg/ml) versus 1.92 pg/ml (0.87–2.43 pg/ml), *p* < 0.001; and TNF-α, 9.06 pg/ml (3.92–83.4 pg/ml) versus 5.35 pg/ml (3.12–7.4 pg/ml), *p* = 0.04. On the other hand, the study did not show meaningful differences between AML patients compared to healthy volunteers for TRAIL: 68.54 pg/ml (19.1–104.8 pgml) versus 79.9 pg/ml (55.4–108.2 pg/ml), *p* = 0.12 (Table 2). Moreover, none of the analyses exhibited any marked differences in the concentration of cytokines according to the WHO classification for all studied cytokines (*p* > 0.05).

**Table 2:** The median (range) values of chosen parameters of AML patients and healthy volunteers

Parameters	No. of patients				
	New diagnosed patients *n* = 76	Healthy volunteers *n* = 40	Patients after an induction treatment		
			After the treatment *n* = 71	With CR *n* = 44	With NR *n* = 27
TRAIL [pg/ml]	68.54 (19.1–104.8)	79.9* (55.4–108.2)	56.52 (19.3–158.8)	74.2 (35.22–158.8)	50.56*** (19.3–88.28)
BAFF [pg/ml]	3615.1 (983.9–9151.4)	651.4* (362.9–1122.3)	3058.1** (972.3–8923.4)	1885.1 (972.3–7645.4)	5036.4*** (1538.2–8923.4)
APRIL [ng/ml]	5.96 (1.65–43.4)	1.68* (1.0–7.56)	5.56 (1.4–69.9)	3.42 (1.4–19.2)	8.89*** (2.1–69.9)
TNF [pg/ml]	9.06 (3.92–83.4)	5.35 * (3.12–7.4)	8.62** (3.8–69.1)	8.51 (3.8–43.1)	9.13 (3.9–69.1)
IL-6 [pg/ml]	12.12 (1.96–43.4)	1.92* (0.87–2.43)*	8.64** (0.57–155.4)	5.42 (0.57–20.1)	19.4*** (4.57–155.4)

The values are presented as median (range)

*AML* acute myeloid leukaemia, *BAFF* B cell-activating factor, *APRIL* a proliferation-inducing ligand, *TRAIL* TNF-related apoptosis-inducing ligand, *TNF* tumour necrosis factor, *IL-6* interleukin 6, *CR* complete remission, *NR* non-responders

*p < 0.05 between AML patients and healthy volunteers

**p < 0.05 between before and after treatment AML patients

***p < 0.05 between patients with CR and NR

In addition, the concentration of BAFF and IL-6 in the group of newly diagnosed patients differed sharply from that of patients after chemotherapy, for BAFF, *p* = 0.02, and IL-6, *p* = 0.0001, respectively. Pronounced differences were observed also between the subgroup of patients with CR and NR. The concentrations were found to be lower in the former, for BAFF, 1,885.1 pg/ml (972.3–7,645.4 pg/ml) versus 5,036.4 pg/ml (1,538.2–8,923.4 pg/ml); IL-6, 5.42 pg/ml (0.57–20.1 pg/ml) versus 19.4 pg/ml (4.57–155.4 pg/ml), *p* = 0.001; and for APRIL, 3.42 ng/ml (1.4–19.2 ng/ml) versus 8.89 ng/ml (2.1–69.9 ng/ml), *p* = 0.04, but not for TNF-α, *p* = 0.23. Conversely, the concentration of TRAIL in the CR subgroup was meaningfully higher compared to the NR one: 74.4 pg/ml (35.22–158.8 pg/ml) versus 50.56 pg/ml (19.3–88.28 pg/ml), *p* = 0.02 (Table 2).

### Correlation with clinical and laboratory data

We subsequently evaluated the association of each cytokine under scrutiny with a series of clinical and haematological variables of well-known parameters of prognosis and tumour load in AML (presented in Table 1). The study did not establish significant differences within the subgroup of patients with different types of cytogenetic risk (favourable vs intermediate I and II vs unfavourable), *p* > 0.05 except for the concentration of TRAIL. The median concentration of TRAIL in the favourable cytogenetic risk group, the intermediate I plus II and unfavourable group was established, standing at 77.1 pg/ml (43.3–104.8 pg/ml), 52.1 pg/ml (22.3–92.3 pg/ml) 5 and 45.6 pg/ml (19.1–88.3), *p* = 0.03, respectively. More importantly, the study did not establish significant differences in concentrations connected with the presence of internal tandem duplications of FLT3 mutations, *p* > 0.05. Statistic analyses were not done for NMP1 and CEBPA due to a small number of patients with positive mutations.

Furthermore, the study demonstrated statistically significant correlations between the concentration of TNF-α, BAFF and TRAIL versus the WBC counts (rho = 0.33, *p* = 0.04 and rho = 0.58, *p* < 0.001 and rho = −0.56, *p* < 0.001, respectively). It also revealed a significantly positive correlation between the concentration of TNF-α and BAFF versus neutrophil counts (rho = 0.33, *p* = 0.04, rho = 0.47, *p* < 0.001) and a negative one with TRAIL (rho = −0.35, *p* = 0.01).

In addition, the analyses showed a meaningful positive correlation between monoblastic cell counts and the concentration of BAFF (rho = 0.37, *p* = 0.01) and a negative one with TRAIL (rho = −0.42, *p* = 0.007). Even more importantly, the study revealed a positive correlation between the concentration of APRIL and the counts of blastic cells in a bone marrow smear (rho = 0.43, *p* = 0.001 and a negative one between the concentration of TRAIL and blastic cell counts in peripheral blood (rho = −0.41, *p* = 0.002) and LDH (rho = −0.52, *p* < 0.001).

What is more, the concentration of BAFF was found to correlate significantly and positively with the concentration of APRIL (rho = 0.53, *p* < 0.001) and TNF (rho = 0.42, *p* = 0.002) and negatively with TRAIL (rho = −0.43, *p* = 0.001). We also established a meaningful negative correlation between TRAIL and IL-6 versus TNF-α (for both rho = −0.31, *p* = 0.04) and a positive one between IL-6 and BAFF (*p* = 0.02). There was, however, no correlation between IL-6 and APRIL (*p* > 0.05).

### Prognostic impact of cytokines on overall survival

**Fig. 1:**
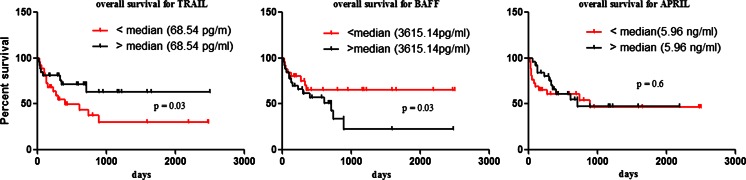
Kaplan–Meier curves of overall survival estimates according to TRAIL, BAFF and APRIL serum levels, in patients with newly diagnosed acute myeloid leukaemia. Patients with TRAIL values higher (*upper or black line*) than median (38.54 pg/ml) have a significant longer OS than patients with lower TRAIL value (*bottom line or red line*). The *two curves* are significantly different (*p* = 0.03). Patients with BAFF values higher (*bottom or black line*) than median (3,615.14 pg/ml) have a significant shorter OS than patients with lower BAFF value (*upper line or red line*). The *two curves* are significantly different (*p* = 0.03). There were no differences of OS between patients with higher values of APRIL than median (5.96 ng/ml) compared to lower values, *p* = 0.6

In the AML group, the favourable cytogenetic risk subgroup is usually excluded from the analysis due to its different biology and outcome. However, there was no change in the value of all the cytokines, except for TRAIL, in predicting survival after these patients had been excluded. Additionally, we observed that pre-treatment AML patients with serum BAFF values higher than the median (3,615.14 pg/ml) had a significantly shorter overall survival (OS) than patients with a lower BAFF value, *p* = 0.03 (Fig. 1). Furthermore, we found that pre-treatment AML patients with serum TRAIL values lower than the median (68.54 pg/ml) had a significantly shorter OS than patients with a higher TRAIL value, *p* = 0.03 (Fig. 1). In the subgroups of APRIL patients, there were no statistically significant differences in the median values (5.96 ng/ml) of APRIL, *p* = 0.61 with regard to OS. As expected, cytogenetic turned out to be a strong predictor of survival in this group of patients (*p* < 0.001). By contrast, LDH, age and WBC were not found to be predictors of survival. Multivariate Cox proportional hazard model, which incorporates all significant factors along with the studied cytokines, showed that only TNF-α and BAFF can be considered independent risk factors of cytogenetic, performance status or age (Table 3).

**Table 3:** Univariate (unadjusted) and multivariate (adjusted) analysis of associations between concentrations of TNF-α superfamily and overall survival of AML patients

	Overall survival		
	HR	95 % confidence interval	*p* Value
All patients			
Univariate analysis			
Sex (male vs female)	1.1612	0.51–2.65	0.71
Age (<60 vs 60+)	1.5322	0.66–3.58	0.32
Cytogenetics			
Favourable vs adverse	5.7120	1.91–0.15.9	0.0005
Intermediate vs adverse	3.7411	1.59–11.64	0.0040
TNF-α (low vs high)	3.4801	1.36–8.91	0.0009
APRIL (low vs high)	0.8110	0.35–1.84	0.6133
BAFF (low vs high)	2.1711	0.87–4.94	0.0319
TRAIL (low vs high)	0.4667	0.19–1.10	0.0369
Multivariate analysis			
TNF-α (low vs high)	2.6917	1.05–5.89	0.0482
BAFF (low vs high)	2.5332	1.09–4.98	0.0502
TRAIL (low vs high)	0.7119	0.29–1.72	0.2912
Patients without favourable prognostic factors: inv(16) and t(8;21)			
Univariate analysis			
Sex (male vs female)	1.1708	0.50–2.72	0.701
Age (<60 vs 60+)	1.3520	0.55–3.27	0.512
Cytogenetics			
Intermediate vs adverse	3.9751	1.73–13.03	0.0030
TNF-α (low vs high)	2.9247	1.17–5.58	0.0218
APRIL (low vs high)	0.8847	0.38–2.05	0.7745
BAFF (low vs high)	2.4225	0.98–5.99	0.0307
TRAIL (low vs high)	0.5780	0.22–1.410	0.0304
Multivariate analysis			
TNF-α (low vs high)	2.6702	1.00–6.75	0.0405
BAFF (low vs high)	2.5365	0.99–6.03	0.0501
TRAIL (low vs high)	0.7092	0.27–1.61	0.3332

Low or high superfamily of TNF refers to values either lower or higher than median observed in all AML patients at the time of diagnosis

*HR* hazard rate, *AML* acute myeloid leukaemia, *BAFF* B cell-activating factor, *APRIL* a proliferation-inducing ligand, *TRAIL* TNF-related apoptosis-inducing ligand, *TNF* tumour necrosis factor, *IL-6* interleukin 6

## Discussion

Over the last few decades, countless attempts have been made at establishing prognostic markers capable of discriminating high-risk patients and at identifying a new complex network of cytokines that either promote or inhibit AML cell growth, progression and the development of drug resistance or sensitivity. Among these cytokines, TRAIL, a member of the TNF family, has emerged as a prominent biologically targeted anti-tumour protein by virtue of its remarkable ability to induce apoptosis in a variety of human cancer cell lines without affecting normal cells [8]. In recent years, TRAIL has raised hopes for its therapeutic potential as an anti-neoplastic agent in different types of tumours, including haematological malignancies, such as AML [30]. The in vitro cytotoxic response of AML cell lines to recombinant TRAIL varies from good to moderate [31, 32]. However, a number of in vitro studies have convincingly shown AML primary cells to be resistant to the proapoptotic activity of TRAIL used as a single agent, despite the presence and functioning of the TRAIL death pathway. Lack of sensitivity to the TRAIL apoptotic pathway can be explained by different mechanisms involving reduced expression of the death receptors (only a minority of the AML cells express TRAIL-R1 and TRAIL-R2) [33]. On the other hand, it has been recently demonstrated that TRAIL sensitivity of AML blasts could be increased by co-treatment with a cytotoxic drug even those who are refractory to conventional chemotherapy [33, 34]. Indeed, Chamuleau et al. confirmed the anti-apoptotic function of decoy TRAIL-R3 in vitro by treating leukaemic cell lines and fresh primary AML samples with recombinant soluble TRAIL. They have provided new evidence indicating that direct targeting of proapoptotic receptors may be an option in the treatment of AML patients. Even more importantly, they established that high expression of the decoy receptor R3 has impact on poor clinical outcome in AML [35].

To the best of our knowledge, there is no data describing the concentration of TRAIL in AML patients or its possible application on biological and clinical features of AML. In this respect, our study revealed significantly lower concentrations of soluble TRAIL in 76 AML patients compared to healthy volunteers. Second, the data showed that the concentration of TRAIL following the administration of induction therapy in the subgroup of CR was meaningfully higher compared to NR and nearly equalled the value in healthy volunteers. Even more importantly, it became clear that TRAIL concentration correlated negatively with risk factors, such as high WBC count and LDH, both of which have prognostic values and are associated with adverse outcomes in AML. Furthermore, based on the obtained data, we found a relevant negative correlation between the concentration of TRAIL and blastic cell counts, as well as meaningful differences within the range of cytogenetic risk groups, since the concentration in patients belonging to the favourable risk group was significantly higher compared to the rest, nearly equaling the values in healthy volunteers. It must be noted that blocking anti-TRAIL antibodies markedly reduced or completely inhibited hypoxia-induced apoptosis in AML1/ETO and that TRAIL is a key regulator of hypoxia-induced apoptosis in these cells [36]. All these established correspondences suggest that decreased concentration of TRAIL can be considered as a high-risk factor in patients with AML. Furthermore, the study found a negative correlation between monoblastic cell counts and TRIAL. Indeed, the dichotomous effect of TRAIL on malignant cells (early induction of apoptosis and monocytic maturation of the surviving cells) might have important therapeutic implications for the treatment of AML [11]. Moreover, in univariate analysis, the data showed that pre-treatment AML patients with serum TRAIL values higher than the median had a significantly shorter OS than patients with a lower TRAIL. The results are not surprising, since the data established also a negative correlation between TRAIL and TNF-α concentration, whose increased serum level is an adverse prognostic factor for survival in AML patients [5]. Summarising the obtained results, we hypothesized that myeloid leukaemic blasts may be principally sensitive to TRAIL-mediated killing by mTRAIL and/or sTRAIL expressed and secreted by naturally effector immune cells as a part of immune surveillance.

BAFF, a glycoprotein belonging to the TNF family, is originally expressed as a type II membrane-bound protein, subsequently cleaved at a putative furin consensus site and released as a soluble protein [37], playing crucial roles in B cell homeostasis, tolerance and malignancy. It must be noted that the regulation of the expression and synthesis of BAFF by IL-10 and IFN-γ at the transcriptional level has also been demonstrated in human promyelocytic leukaemia cell cultures [38]. On the other hand, IL-6 and TNF-a may induce IL-10 secretion with the potential to inhibit IL-6, TNF-α production, which represents an important amplification loop of the inflammatory response [5]. Changes in one of these cytokines due to a malignancy lead to compensatory mechanisms that may alter the cytokine network.

The anti-apoptotic activity of APRIL has so far been demonstrated in B lymphoma, multiple myeloma and B-CLL cells, suggesting a general oncogenic role for APRIL in haematological malignancies. Recently, Boci et al. demonstrated by means of the Western blotting analysis that exogenous expression of APRIL upregulated Bcl-2 in CD34+ cells, whereas APRIL neutralisation resulted in Bcl-2 downregulation in primary AML cells, indicating that APRIL protects AML from chemotherapeutic drugs through upregulation of Bcl-2 [26]. Conversely, blocking the function of APRIL by recombinant soluble APRIL receptors increased the chemotherapeutic drug-induced cell death in AML cells. These results indicate that APRIL acts in an autocrine fashion to protect AML cells from drug-induced death and foresee a therapeutic potential of APRIL antagonists in the treatment of AML.

Our study has been the first to have established the concentration of BAFF and APRIL in AML patients, since BAFF and APRIL are not only a transmembrane proteins but it also exist in soluble forms derived from the intracellular cleavage of the full-length protein and confirmed their higher values compared to healthy volunteers. More importantly, we have revealed a meaningful decrease in the concentration of BAFF not APRIL following the administration of induction chemotherapy. We further extended this observation by demonstrating concentrations of APRIL and BAFF to be significantly lower in the subgroup of patients with CR compared to those who did not respond to therapy. Furthermore, we found a correlation between both proteins, and most importantly, between concentration of BAFF and the growth factor IL-6, a pleiotropic cytokine with multiple biological activities in vitro and in vivo, including human myeloid cell proliferation [39]. Since serum levels of IL-6 may reflect the activity of AML, it is possible that the elevated serum BAFF concentrations found in untreated patients and statistically higher in active NR, compared to patients with CR, may be related to the growth of myeloid cells. More importantly, the study showed that concentration of BAFF not APRIL was positively associated with WBC count and TNF-α concentration at diagnosis, what suggest likewise that BAFF is closely related to the severity and prognosis of the disease. In addition, the study revealed a positive correlation between the concentration of APRIL and the counts of blastic cells in a bone marrow smear, and a negative one with the concentration of TRAIL, which was shown to be a prognostic factor for OS in AML patients. Those do not come as a surprise since BAFF and APRIL are a well-known stimulator factors for B cell homeostasis and survival through the activation of NF-κB pathway. Indeed, there is ever more evidence that this pathway activity is also observable in the CD34^+^ AML cells [22]. Given that NF-κB activity is not restricted to specific AML subtypes or genetic abnormalities, it is possible that the signalling is universally essential for myeloid leukaemia progression.

Furthermore, our analyses established important differences with regard to OS in subgroups with the cut-off point as the median of BAFF but not APRIL concentration, since the patients with a higher concentration had a significantly shorter OS. Our results fall in line with a study conducted by Kim et al., who showed in multivariate analysis that serum BAFF but not APRIL was an independent prognostic factor for OS and progression-free survival in NHL patients [40]. Therefore, it is reasonable to presume that APRIL and BAFF may play overlapping and synergetic roles in the pathogenesis of AML. In fact, it has been reported that BAFF and APRIL can associate with each other to form a heterotrimer capable of stimulating B cell proliferation in patients with autoimmune diseases [41]. Whether this heterotrimer exists and plays a part in AML needs further investigation.

In conclusion, our results have demonstrated that serum concentrations of all TNF superfamily could constitute a useful biomarker of AML disease activity and progression. Pre-treatment concentrations of BAFF and TRAIL could also serve as a prognostic factor for OS. The ligands may therefore be a novel therapeutic target in AML.
